# Feed intake, nutrient digestibility, and selected rumen parameters in feedlot bulls fed diets with different feed additives

**DOI:** 10.1371/journal.pone.0259414

**Published:** 2021-11-02

**Authors:** Breno de Castro Silva, Marcos Vinicius Carneiro Pacheco, Letícia Artuzo Godoi, Gilyard Angelo Pinheiro de Souza, Nathália Veloso Trópia, Pauliane Pucetti, Flávia Adriane de Sales Silva, Ana Clara Baião Menezes, Luciana Navajas Rennó, Mário Fonseca Paulino, Jon Patrick Schoonmaker, Sebastião de Campos Valadares Filho

**Affiliations:** 1 Department of Animal Science, Universidade Federal de Viçosa, Viçosa, Minas Gerais, Brazil; 2 Department of Animal Sciences, Center for Nutrition and Pregnancy, North Dakota State University, Fargo, North Dakota, United States of America; 3 Department of Animal Science, Purdue University, West Lafayette, Indiana, United States of America; Tokat Gaziosmanpasa Universitesi, TURKEY

## Abstract

An experiment was conducted to evaluate the feed intake, nutrient digestibility and selected rumen parameters in feedlot bulls fed diets containing different feed additives. Six rumen-cannulated Nellore bulls (age = 8 ± 1.0 months; initial BW = 225 ± 13.2 kg) were distributed in a 6 × 6 Latin square design. Six experimental diets based on 30% corn silage and 70% concentrate on a dry matter (DM) basis were evaluated. Diets differed in feed additive on a DM basis, as follows: 1.4% bicarbonate and magnesium oxide in 3:1 ratio (BOX); 36 ppm lasalocid sodium (LAS); 30 ppm monensin sodium (MON); 25 ppm virginiamycin (VIR); 30 ppm monensin sodium plus 25 ppm virginiamycin (MV); and 3.15% commercial mineral supplement containing D-limonene and exogenous α-amylase (EOA). The experiment lasted 144 d, with six periods of 24 d. Each period consisted of 14 d for dietary adaptation, 3 d for feces and urine collection, and 7 d for omasal and ruminal digesta collection. Bulls fed the BOX diet showed greater (*P* < 0.05) intake of DM, organic matter (OM), neutral detergent fiber (apNDF), crude protein (CP), and starch compared to the other diets. Diets with LAS, MON, VIR, MV, or EOA did not influence (*P* > 0.05) the DM, OM, apNDF, CP, or starch intake of feedlot bulls. Bulls fed the EOA diet showed greater (trend; *P* = 0.09) ruminal digestibility of starch compared to the other diets. The feed additives did not affect (*P* > 0.05) the intestinal or total tract digestibility of starch, rumen pH, microbial efficiency, total rumen fluid, dilution rate, rate of intake, rate of degradation, or passage rate of the DM, OM, apNDF, and starch. In conclusion, LAS, MON, VIR, MV, and EOA diets reduced nutrient intake compared to BOX. Although all feed additives presented similar effects on rumen pH, temperature, and kinetics the presence of exogenous α-amylase in the EOA diet may increase ruminal starch digestibility and apparent total tract digestibility of DM and OM.

## Introduction

Feed additives (e.g., buffers, ionophores, non-ionophore antibiotics, plant compounds with antibacterial properties, exogenous enzymes, etc.) have been increasingly used worldwide to improve feed efficiency or to benefit the ruminal health, especially when greater animal growth performance is required, such as in feedlot systems [1–3]. Recent surveys [2, 3] showed that more than 92% of the feedlots in Brazil and the United States use some type of feed additive in their receiving and finishing diets.

Buffers, such as sodium bicarbonate, were commonly used in feedlot diets in the past and are still an option to mitigate the occurrence of metabolic disorders and to increase intake and daily gain [4–6]. However, sodium bicarbonate is a soluble buffer, which is short-lived in the rumen and its use as a sole additive may not effectively buffer the continued acid production in the rumen [7]. Thus, a combination of buffers and alkalizers (e.g., magnesium oxide) in feedlot diets have been studied [8–10], and additive effects on productive parameters are reported [9].

Over the years, buffers and alkalizers have been replaced by ionophores [e.g., monensin sodium (MON) and lasalocid sodium (LAS)] and non-ionophore antibiotics [e.g., virginiamycin (VIR)] in feedlot diets. The dietary inclusion of ionophores and non-ionophores as sole feed additives or in combination has increased propionic acid production, improved feed efficiency, and reduced the incidence of metabolic disorders compared to diets containing no additives [11, 12]. However, plant compounds with antibacterial properties and exogenous enzymes have been increasingly used to replace antibiotics in feedlot diets [13, 14]. In this context, studies [13, 14] have shown that animals fed diets containing essential oils plus exogenous α-amylase (EOA) may show increased feed intake, starch digestibility, and average daily gain compared to those fed diets with other feed additives, such as MON.

Although there are benefits to animals fed with feed additives compared to those fed none, the responses when different feed additives are compared have not been consistent. For example, according to NASEM [15], the use of buffers in cattle diets has variable results, and the answers obtained often do not justify the costs of its adoption. Studies [16–18] showed that both MON and VIR as sole feed additives or in combination (MV) may improve cattle productive parameters. Also, animals fed diets containing VIR seem to present greater feed intake compared to those fed diets with ionophores [16]. However, other studies [19–21] did not verify differences in feed intake, performance or digestive parameters when MON and VIR were provided to feedlot cattle as sole additives or in combination.

The variability in experimental conditions (e.g., dose applied, days on feed, sex, breed, age, etc.) makes the comparison of different studies difficult. Therefore, a greater number of feed additives evaluated in a unique study may allow direct comparison and a better understanding of the real effects of such additives. We hypothesized that: 1) bulls fed bicarbonate/magnesium oxide in 3:1 ratio (BOX) or EOA diets would exhibit greater intake compared to those fed diets with LAS, MON, VIR, or MV; and 2) bulls fed an EOA diet would present greater ruminal and total tract digestibility of starch compared to animals fed diets with BOX, LAS, MON, VIR, or MV. Thus, the objective of this study was to evaluate the feed intake, nutrient digestibility and selected rumen parameters in feedlot bulls fed diets containing different feed additives.

## Material and methods

The experiment was conducted in the Experimental Feedlot of the Animal Science Department at the Universidade Federal de Viçosa (Viçosa, MG, Brazil). All procedures involving animal care and management were approved (protocol number 22/2017) by the Ethics Committee for Animal Use of the Universidade Federal de Viçosa.

### Animals, experimental design, facilities, and diet

Six rumen-cannulated Nellore bulls (age = 8 ± 1.0 months; initial body weight = 225 ± 13.2 kg) sourced from a Universidade Federal de Viçosa herd in Viçosa, Minas Gerais were distributed in a 6 × 6 Latin square design balanced for residual effects. Initially, the animals were identified, treated for internal and external parasites and, housed in a tie-stall barn with a concrete floor and equipped with water and feed troughs. Thirty days prior the beginning of the experiment, the rumen cannulation was performed in a one-stage operation as described by Girard et al. [22]. An incision was made in the skin of the left flank, in the dorsoventral direction, approximately 5 cm ventral to the transverse process of the lumbar vertebrae, and 8 cm caudal to the 13th rib. Muscle layers (external oblique, internal oblique, and transverse abdominal muscles) were divulsioned in the direction of the fibers allowing peritoneum access. The rumen wall was exposed, and then rumen wall, peritoneum, muscles, and skin were sutured together. A dorsoventral incision was made into the center of the exposed rumen wall, in which was inserted a silicone rumen cannula (Kehl Industria e Comércio Ltda ME, São Carlos, São Paulo, Brazil).

**Fig 1:**
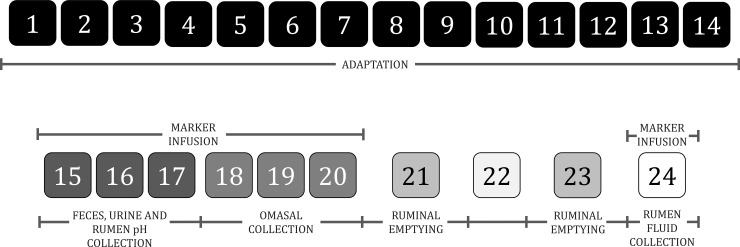
Experimental period scheme.

The experiment lasted 144 d, in six 6 periods of 24 d. Each period consisted of 14 d for dietary adaptation [23] and 10 d for data collection (Fig 1). It is worth mentioning that during the dietary adaptation periods, the bulls were allocated individual pens (2 × 20 m) for exercise. At the end of the dietary adaptation period, they were allocated back to the tie-stall pens.

Six experimental diets based on 30% corn silage and 70% of concentrate (DM basis) were evaluated. The diets differed in feed additive on a DM basis, as follows: 1.4% bicarbonate and magnesium oxide in 3:1 ratio (BOX); 36 ppm lasalocid sodium (LAS; Taurotec^®^, Zoetis Inc., Campinas, São Paulo, Brazil); 30 ppm monensin sodium (MON; Bovensin^®^, Phibro Animal Health, Guarulhos, São Paulo, Brazil); 25 ppm virginiamycin (VIR; V-MAX^®^ 2, Phibro Animal Health, Guarulhos, São Paulo, Brazil); 30 ppm monensin sodium plus 25 ppm virginiamycin (MV); 3.15% commercial mineral supplement with D-limonene and exogenous α-amylase (EOA; Fosbovi^®^ Confinamento CRINA^®^ RumiStar™, DSM S.A., Mairinque, São Paulo, Brazil). The diets were formulated according to BR-CORTE recommendations [24] to provide approximately 12.7% CP on a DM basis and support an average daily gain of approximately 1.2 kg/d (Table 1).

**Table 1:** Feedstuffs and chemical composition of experimental diets.

Item	BOX	LAS	MON	VIR	MV	EOA
Feed, % of dry matter						
Corn Silage	30.00	30.00	30.00	30.00	30.00	30.00
Finely ground corn	47.00	47.00	47.00	47.00	47.00	47.00
Wheat bran meal	15.90	15.90	15.90	15.90	15.90	14.95
Soybean meal	4.00	4.00	4.00	4.00	4.00	4.00
Urea + AS^1^	0.90	0.90	0.90	0.90	0.90	0.90
Salt	0.20	0.20	0.20	0.20	0.20	-
Limestone	0.50	0.50	0.50	0.50	0.50	-
Micromineral supplement^2^	0.10	0.10	0.10	0.10	0.10	-
Sodium bicarbonate	1.05	-	-	-	-	-
Magnesium oxide	0.35	-	-	-	-	-
Sodium lasalocid	-	0.024	-	-	-	-
Sodium monensin	-	-	0.015	-	0.015	-
Virginiamycin	-	-	-	0.125	0.125	-
Fosbovi^®^ CRINA^®^ RumiStar™^3^	-	-	-	-	-	3.15
Silica	-	1.38	1.39	1.28	1.27	-
Dry matter, % as fed	58.64	58.63	58.61	58.64	58.62	58.58
Chemical composition, % of dry matter						
Organic matter	94.40	94.40	94.40	94.40	94.40	93.93
Crude protein	12.75	12.75	12.75	12.75	12.75	12.65
Ether extract	4.10	4.10	4.10	4.10	4.10	4.07
Neutral detergent fiber^4^	25.70	25.70	25.70	25.70	25.70	25.56
Starch	47.37	47.37	47.37	47.37	47.37	47.05
Non-fiber carbohydrates^5^	53.36	53.36	53.36	53.36	53.36	53.19

Diet with bicarbonate and magnesium oxide (BOX); Diet with lasalocid sodium (LAS; Taurotec^®^, Zoetis Inc., Campinas, São Paulo, Brazil); diet with monensin sodium (MON; Bovensin^®^ 200, Phibro Animal Health, Guarulhos, São Paulo, Brazil); diet with virginiamycin (VIR; V-MAX^®^ 2, Phibro Animal health, Guarulhos, São Paulo, Brazil); diet with monensin sodium and virginiamycin (VM); and diet with essentials oleo and exogenous α-amylase (EOA; Fosbovi^®^ Confinamento CRINA^®^ RumiStar™, DSM Produtos Nutricionais Brasil S.A., Mairinque, São Paulo, Brazil).

^1^ Urea + ammonium sulfate in a 9:1 ratio.

^2^ The micromineral supplement was composed of 56.3% zinc sulfate, 26.2% manganese sulfate, 16.8% copper sulfate, 0.4% potassium iodide, 0.2% cobalt sulfate, and 0.1% sodium selenite.

^3^ Supplement guarantees (per kg of DM): 140–180 g of Ca, 8 mg of Co (Min), 6.7 mg of Cr (Min), 540 mg of Cu (Min), 36 g of S (Min), 160 mg of F (Max), 16 g of P (Min), 27.5 mg of I (Min), 20 g of Mg (Min), 1070 mg of Mn (Min), 6.7 mg of Se (Min), 56 g of Na (Min), 160 mg of F (Max), 2,000 mg of Zn (Min), 1,140 mg of D-limonene, and 11,400 KNU of α-amylase.

^4^ Neutral detergent fiber corrected for residual ash and residual nitrogenous compounds.

^5^ Non-fiber carbohydrates = 100 − [(crude protein–crude protein from urea + urea) + neutral detergent fiber corrected for residual ash and residual nitrogenous compounds + ether extract + ash].

Corn silage and concentrate feed were weighed separately, hand-mixed at the time of feeding and provided twice per day at 08:00 and 16:00 h as a total mixed ration. Bulls had free access to clean water and were allowed 5% refusals on an as-fed basis. Once per week, corn silage was sampled and dried in a non-ventilated oven at 105°C for 16 h to adjust the diet DM content.

### Intake, total tract digestibility, and microbial efficiency

The weights of forage and concentrate feeds offered, as well as refusals were recorded daily during the collection period (from d 15 to 24). Approximately 400 g (as-is basis) of all diet ingredients and refusals were sampled daily and stored at –20°C during the data collection period (from d 15 to d 24). At the end of each collection period, all samples were dried in a ventilated oven (55 ˚C) for 72 h and ground in a knife mill (Tecnal, Piracicaba, São Paulo, Brazil) with a 1-mm sieve. Daily samples of each diet ingredient were grouped equally (DM basis) in subsamples of 250 g for each period. Also, daily refusal samples were grouped in subsamples of 250 g for each animal within a period. The proportion of daily refusal in the composite sample was based on the amount of refusal verified daily on a DM basis divided by the total amount of refusals verified during the collection period. The samples were packed in plastic bags for further laboratory analyses. The ingredient samples were analyzed individually and used to calculate dietary composition.

From d 15 to d 17 of the experimental period, 24 h fecal and urine outputs were determined for all bulls, as described by Silva et al. [25]. Feces were collected from droppings on the concrete floor and placed in 30 L buckets. At the end of each collection day (24 h), the buckets containing the samples were weighed. Feces were hand-mixed for 5 min to ensure homogeneity. Subsamples of approximately 250 g were collected, dried in a forced-air oven at 55°C for 72 h and ground in a knife mill with a 1-mm sieve. A composite sample of approximately 50 g was obtained for each animal a within period, based on the DM content of the feces collected each day. The samples were stored in plastic bags for further laboratory analyses. Urine output was collected from d 15 to d 17 (over a 24-h period) using collecting funnels attached to bulls with hoses to carry the urine to 20-L containers with 200 mL of 50% sulfuric acid (v/v). At the end of the first collection day, the total urine volume was measured and homogenized manually. Then, subsamples of 50 mL were collected and immediately frozen at –20 ˚C. The previously added acid solution (200 mL) was subtracted from the total urine volume. During the subsequent collection days, the same procedures for the collection and measurement of urine volume were adopted. However, the collected subsample was proportional to the urine volume obtained on the first day. After each collection day, urine subsamples were combined with the samples from the previous days and immediately frozen at –20 ˚C. So, one urine sample was obtained for each animal within each period.

### Rumen pH evaluation

Rumen pH was evaluated for all bulls over a 72-h period (d 15 to d 17 of each experimental period). The readings were performed every 15 min using an intra-ruminal bolus pH meter (Model: WellCow^TM^; Roslin, UK). Rumen status was classified into three categories: acute acidosis (rumen pH below 5.2 for more than 6 h per day), subacute acidosis (ruminal pH below 5.6 for more than 12 h per day), and normal (rumen pH above 5.6 for more than 12 h per day) [26]. The area under the curve and duration time of rumen pH were evaluated for each interval.

### Marker infusion and partial digestibility estimation

From d 15 to d 20 of the experimental period, 5 g of Co-EDTA (680 mg Co) [27] were infused daily via ruminal cannula. The Co-EDTA was diluted in distilled water, and the infusion was performed with the aid of a peristaltic pump (BP-600.4; Milan Equipamentos Científicos, Colombo, Paraná, Brazil) at a rate of 115 mL h^–1^. Starting on d 18 of each period, eight omasal digesta samples were collected every 9 h. Omasal digesta were collected at 08:00 and 17:00 h (d 18), 02:00, 11:00 and 20:00 h (d 19), 05:00, 14:00, and 23:00 h (d 20). Omasal digesta collection via ruminal cannula was performed as described by Huhtanen et al. [28] and adapted by Leão [29]. At each omasal digesta collection time, 200 mL of omasal digesta was filtered (porosity of 100 μm, 44% of surface, Sefar Nitex 100/44, Sefar, Thal, Switzerland). Subsamples of solid and liquid plus small-particle phases were individually lyophilized (Liobras, São Carlos, São Paulo, Brazil) and ground in a knife mill with a 1-mm sieve. Then, subsamples of solid phase from eight collection times were combined into one subsample. The same process was carried out for eight subsamples of liquid plus small-particle phase. Therefore, one solid phase and one liquid plus small-particle phase sample were obtained for each animal within period.

### Ruminal emptying

The rate of intake and ruminal *pool* for each diet were estimated using the technique described by Allen and Linton [30]. On d 21 the rumen was completely emptied immediately before the morning diet was provided. Also, on d 23 the rumen was completely emptied 4 h after the morning diet was provided. After emptying the rumen, the digesta was weighed and filtered through a double layer of cheesecloth for separation of solid and liquid fractions, which were weighed. Subsamples of approximately 200 g of solid phase and 200 mL of liquid phase were individually collected. After sampling, the digesta solid and liquid phases were mixed and returned to the rumen of the respective animals. The subsamples were lyophilized and ground in a knife mill with a 1-mm sieve. Rumen digesta from both emptying procedures were reconstituted into a unique sample by combining the subsamples of solid and liquid phases. Reconstitution was based on the amount of solid and liquid (DM basis) verified in each emptying. Then, reconstituted rumen digesta were stored in plastic bags for further laboratory analyses. Therefore, one rumen digesta sample was obtained for each animal within period.

### Passage rate of rumen fluid and total rumen fluid

On d 24 of each period, immediately before the morning feeding, a 250-mL rumen digesta sample was withdrawn from eight different points after hand mixing of ruminal contents (0 h), and an intraruminal dose of 250 mL of Co-EDTA solution (680 mg Co) [27] was administered and mixed with rumen contents. At 1, 2, 3, 4, 6, 8, 10, 12, 18, and 24 h post-dosing, approximately 250 mL of rumen digesta was taken. At each rumen digesta collection time, the whole sample was filtered (porosity of 100 μm, 44% of surface, Sefar Nitex 100/44, Sefar, Thal, Switzerland). Then, 50 mL of rumen fluid was sampled and frozen at –20°C for further cobalt content evaluation.

### Chemical analyses

Samples of feedstuff, refusals, feces, and omasal digesta were analyzed for DM, ash, N, and ether extract (EE), according to AOAC [31] method numbers 934.01, 930.05, and 981.10 and AOAC [32] method number 945.16, respectively. The OM content was determined as the difference between DM content and ash content. Crude protein content was calculated by multiplying the total nitrogen content by 6.25. The neutral detergent fiber (NDF) was evaluated according to Mertens et al. [33], without the addition of sodium sulfite and with the addition of thermostable α-amylase. The NDF content was corrected for residual ash and protein (apNDF). Estimations of neutral detergent insoluble nitrogen (NDIN) followed the technique described by Licitra et al. [34]. The indigestible neutral detergent fiber (iNDF) content was calculated after *in situ* incubation of the samples from three cannulated bulls using F57 bags (Ankom Technology, Macedon, NY, USA) for 288 h, as described by Valente et al. [35] for tropical forages. Non-fibrous carbohydrates were calculated according to Detmann and Valadares Filho [36]. The starch analyses were performed following the recommendations of Silva et al. [37]. The ruminal digesta was analyzed for DM, OM, apNDF, and starch, following the procedures previously described.

Samples of omasal digesta and rumen fluid were analyzed for cobalt by atomic absorption spectrophotometry (Spctr AA-800; Varian spectrometer, Harbor City, CA, USA) according to Kimura and Miller [38]. Urine samples were analyzed for concentrations of uric acid (automated biochemical analyzer, Mindray, Shenzhen, China) and allantoin, according to Chen and Gomes [39]. Microbial efficiency was estimated as described by Barbosa et al. [40] according to the daily purine derivative excretion and calculated from the sum of allantoin and uric acid excretion in urine. Microbial efficiency was expressed as grams of microbial crude protein (MCP) per kilogram of total digestible nutrient intake (TDN).

### Calculations and statistical analysis

The intake of dietary component, such as DM, OM, starch, etc., were estimated by subtracting refusal composition from the total offered. Total intake was then divided by days on feed.

Dry matter and nutrient fluxes in the omasum were estimated using a double-marker system [41, 42]. Indigestible neutral detergent fiber was used as a solid phase marker, and Co-EDTA as a liquid phase marker [42]. Ruminal and intestinal digestibility were expressed in relation to intake for each portion of the digestive tract, as follows:

Ruminaldigestibility=[(intake–omasaldigestaflow)/intake]×100 (1)

Intestinaldigestibility=totaltractdigestibility–rumendigestibility (2)

Totaltractdigestibility=[(intake−outputinfeces)/intake]×100 (3)

The dilution rate, volume of rumen fluid, and flow rate of rumen fluid were estimated according to models described by Czerkawski [43]:

$$C_{t}=C_{0}\times e^{-Dt}$$ (4)

where C_t_ = marker concentration in time “t”, ppm; C_0_ = marker concentration at time zero obtained from the intercept, ppm; D = dilution rate, % h^–1^; and t = time, h.

$$V=Q/C_{0}$$ (5)

where V = rumen fluid volume, L; and Q = amount of marker injected, g.

F=D×V (6)

where F = flow rate of rumen fluid, L h^–1^.

The rates of ingestion (ki), passage (kp), and digestion (kd) were calculated according to the following models:

ki=(intake/rumenpool)×100 (7)

kp=(omasumflow/rumenpool)×100 (8)

kd=ki−kp (9)

where ki = ingestion rate of feed fractions (% h^–1^); intake = feed intake (kg h^–1^); rumen pool = amount of total rumen DM (kg); kp = passage rate of feed fractions (% h^–1^); omasum flow = amount of DM or nutrients in the omasum (kg h^–1^); and kd = digestion rate of diet fractions (% h^–1^).

Data were analyzed using the PROC MIXED procedure of SAS (version 9.4, SAS Institute Inc., Cary, NC, USA) following the general model:

$$Y_{ijkm}=\mu +Diet_{i}+a_{j}+p_{k}+e_{ijkm}$$

where Y_ijkm_ is the observed measurement; μ is the overall mean; Diet_i_ is the fixed-effect of the i^th^ level of dietary treatment (six levels); a_j_ is the random effect of the j^th^ animal (six levels), with a_j_ ~ N(0, σ_a_^2^); p_k_ is the random effect of the j^th^ level of period (six levels), with p_k_ ~ N(0, σ_p_^2^); and e_ijkm_ is the random error associated with Y_ijkm_, with e_ijkm_ ~N(0, σ_e_^2^). Advantages of using random effects under a Latin square experiment are reported by Kononoff and Hanford [44]. Results were assumed as significant when P ≤ 0.05 and “trending” when 0.05 < P ≤ 0.10.

## Results

### Intake, digestibility, and microbial CP

Intake, ruminal, intestinal, and total tract digestibility of DM and OM are shown in Table 2. Bulls fed the BOX diet showed approximately 17.5 and 17.4% greater (*P* < 0.05) DM and OM intake, respectively, compared to those fed the other diets. On the other hand, the DM and OM intake did not differ (*P* > 0.05) between bulls fed diets with LAS, MON, VIR, MV, or EOA.

**Table 2:** The dry matter (DM) and organic matter (OM) intake, ruminal, intestinal, and total tract digestibility of feedlot bulls fed diet with different feed additives.

Item	BOX	LAS	MON	VIR	MV	EOA	SEM	*P*-Value
DM intake, kg/d	6.68^a^	5.74^b^	5.59^b^	5.69^b^	5.69^b^	5.73^b^	0.376	0.040
DM digestion, %								
Rumen	50.26	48.35	50.19	50.04	49.30	52.33	2.532	0.897
Intestines	20.97	23.86	25.04	24.42	24.11	26.40	2.847	0.813
Total tract	71.23^c^	72.21^b^^c^	75.23^b^	74.46^b^^c^	73.41^b^^c^	78.73^a^	1.491	0.012
OM intake, kg/d	6.30^a^	5.43^b^	5.38^b^	5.28^b^	5.37^b^	5.38^b^	0.355	0.038
OM digestion, %								
Rumen	55.71	54.32	56.11	55.68	54.75	57.66	2.496	0.932
Intestines	17.32	20.70	21.69	20.97	21.36	23.36	2.871	0.751
Total tract	73.03^c^	75.02^b^^c^	77.80^b^	76.65^b^^c^	76.11^b^^c^	81.02^a^	1.495	0.006

Diets were composed of 30% corn silage and 70% concentrate on a DM basis. The diets differed in the feed additive on a DM basis, as follows: 1.4% bicarbonate and magnesium oxide in 3:1 ratio (BOX); 36 ppm lasalocid sodium (LAS; Taurotec^®^, Zoetis Inc., Campinas, São Paulo, Brazil); 30 ppm monensin sodium (MON; Bovensin^®^, Phibro Animal Health, Guarulhos, São Paulo, Brazil); 25 ppm virginiamycin (VIR; V-MAX^®^ 2, Phibro Animal Health, Guarulhos, São Paulo, Brazil); 30 ppm monensin sodium plus 25 ppm virginiamycin (VM); or 3.15% commercial mineral supplement with D-limonene and exogenous α-amylase (EOA; Fosbovi^®^ Confinamento CRINA^®^ RumiStar™, DSM S.A., Mairinque, São Paulo, Brazil).

^a,b^ Means with different superscripts in the same row are different (*P* ≤ 0.05).

The feed additives did not affect (*P* > 0.14) ruminal or intestinal digestibility of DM and OM. However, bulls fed the EOA diet presented approximately 6.5 and 6.9% greater (*P* < 0.05) total tract digestibility of DM and OM, respectively, compared to animals fed diets with BOX, LAS, MON, VIR, or MV. Also, bulls fed the MON diet showed approximately 5.3 and 6.1% greater (*P* < 0.05) total tract digestibility of DM and OM, respectively, compared to those fed diets with BOX. However, animal fed diets with LAS, MON, VIR or MV presented similar (*P* > 0.05) total tract digestibility of DM and OM.

Intake, and ruminal, intestinal, and total tract digestibility of starch, apNDF, and CP are presented in Table 3. Bulls fed diets with BOX diet showed greater (*P* < 0.05) intake of starch, apNDF, and CP compared to those fed diets with LAS, MON, VIR, MV, or EOA. On the other hand, starch, apNDF, and CP intake did not differ (*P* > 0.05) between diets with LAS, MON, VIR, MV, or EOA.

**Table 3:** The intake and digestibility of starch, neutral detergent fiber corrected for ash and protein (apNDF), and crude protein (CP), total digestible nutrient (TDN) intake, and microbial efficiency of feedlot bulls fed diet with different feed additives.

Item	BOX	LAS	MON	VIR	MV	EOA	SEM	*P*-Value
Starch intake, kg/d	3.08^a^	2.65^b^	2.63^b^	2.58^b^	2.63^b^	2.62^b^	0.171	0.004
Starch digestion, %								
Rumen	78.43^b^	79.48^b^	81.42^b^	81.32^b^	79.76^b^	83.32^a^	1.129	0.090
Intestines	10.71	9.13	8.11	8.59	9.39	8.72	1.187	0.717
Total tract	89.14	88.61	89.53	89.91	89.15	92.04	1.416	0.514
apNDF intake, kg/d	1.73^a^	1.47^b^	1.46^b^	1.43^b^	1.46^b^	1.47^b^	0.091	0.020
apNDF digestion, %								
Rumen	51.61	52.54	53.29	52.30	50.78	49.99	2.228	0.734
Intestines	7.66	6.19	7.02	6.73	7.02	7.13	0.766	0.840
Total tract	59.27	58.73	60.31	59.03	57.80	57.12	2.214	0.886
CP intake, kg/d	0.85^a^	0.73^b^	0.72^b^	0.71^b^	0.73^b^	0.72^b^	0.048	0.034
CP digestion, %								
Rumen	6.86	1.51	6.04	4.66	7.57	8.85	3.617	0.505
Intestines	64.79	70.01	68.12	68.87	66.37	65.73	4.076	0.828
Total tract	71.65	71.52	74.16	73.53	73.94	74.58	1.236	0.283
TDN intake, kg/d	4.84	4.42	4.56	4.42	4.45	4.73	0.333	0.444
MCP^1^, g/d	610	550	548	562	562	587	51.5	0.710
MCP, g/kgTDN	125	125	121	127	125	124	5.2	0.978
MCP, g/kgCPintake	753	753	756	778	766	812	26.8	0.567

Diets were composed of 30% corn silage and 70% concentrate on a DM basis. The diets differed in the feed additive on a DM basis, as follows: 1.4% bicarbonate and magnesium oxide in 3:1 ratio (BOX); 36 ppm lasalocid sodium (LAS; Taurotec^®^, Zoetis Inc., Campinas, São Paulo, Brazil); 30 ppm monensin sodium (MON; Bovensin^®^, Phibro Animal Health, Guarulhos, São Paulo, Brazil); 25 ppm virginiamycin (VIR; V-MAX^®^ 2, Phibro Animal Health, Guarulhos, São Paulo, Brazil); 30 ppm monensin sodium plus 25 ppm virginiamycin (VM); or 3.15% commercial mineral supplement with D-limonene and exogenous α-amylase (EOA; Fosbovi^®^ Confinamento CRINA^®^ RumiStar™, DSM S.A., Mairinque, São Paulo, Brazil).

^1^Microbial crude protein.

^a,b^Means with different superscripts in the same row are different (*P* ≤ 0.05).

Bulls fed EOA showed approximately 4.1% greater (trend; *P* = 0.09) ruminal digestibility of starch compared to those fed diets with BOX, LAS, MON, VIR, or MV. Besides that, the ruminal digestibility of starch did not differ between diets with BOX, LAS, MON, VIR, or MV. Feed additives did not affect (*P* = 0.71; *P* = 0.51) the intestinal or total tract digestibility, respectively, of starch. There was no effect of feed additives (*P* > 0.28) on the ruminal, intestinal, or total tract digestibility of apNDF and CP.

There was no effect (*P* > 0.44) of feed additive on TDN intake, MCP g/d, MCP g/kgTDN, and MCP g/kgCPintake.

### Digestion kinetics and rumen pH

The feed additives did not affect (*P* ≥ 0.15) the rate of intake, rate of degradation, or rate of passage of DM, OM, apNDF, and starch (Table 4). There were no differences (*P* > 0.16) among the additives for total rumen fluid or dilution rate. However, bulls fed diets with BOX and EOA presented approximately 26.3 and 28.5% greater (*P* = 0.03) flow rate of rumen fluid, respectively, compared to diets with LAS, MON, VIR, or MV.

**Table 4:** The total rumen fluid, dilution rate, flow rate of rumen fluid and rumen kinetics of dry matter (DM), organic matter (OM), neutral detergent fiber corrected for ash and protein (apNDF), and starch of feedlot bulls fed diet with different feed additives.

Item	BOX	LAS	MON	VIR	MV	EOA	SEM	*P*-Value
DM rates, % h^–1^								
rate of intake	10.79	8.79	9.37	9.15	10.00	10.49	1.002	0.365
rate of passage	5.65	5.22	5.16	4.73	5.36	5.08	0.536	0.717
rate of degradation	5.16	3.96	4.19	4.18	4.43	5.67	0.596	0.150
OM rates, % h^–1^								
rate of intake	11.80	10.00	10.36	9.89	11.44	11.51	1.040	0.378
rate of passage	5.27	4.82	4.91	4.73	5.60	4.68	0.565	0.651
rate of degradation	6.40	5.00	5.20	5.20	5.80	6.60	0.663	0.287
NDFap rates, % h^–1^								
rate of intake	4.60	4.00	4.00	4.00	4.60	5.60	0.643	0.284
rate of passage	2.21	1.98	1.79	1.99	2.42	2.81	0.383	0.346
rate of degradation	2.59	2.20	2.21	2.00	2.41	2.60	0.305	0.420
Starch rates, % h^–1^								
rate of intake	25.00	25.20	24.80	20.69	26.81	27.42	2.069	0.846
rate of passage	4.82	4.22	4.22	4.49	4.65	3.59	0.513	0.412
rate of degradation	20.20	20.80	20.60	21.60	22.00	23.80	1.690	0.570
Total rumen fluid, L d^–1^	36.62	33.34	32.59	31.36	33.00	36.11	2.388	0.331
Dilution rate, % h^–1^	12.55	10.76	10.80	11.79	11.39	12.96	1.024	0.163
Flow rate of rumen fluid, L h^–1^	4.60^a^	3.59^b^	3.52^b^	3.70^b^	3.76^b^	4.68^a^	0.441	0.030

Diets were composed of 30% corn silage and 70% concentrate on a DM basis. The diets differed in the feed additive on a DM basis, as follows: 1.4% bicarbonate and magnesium oxide in 3:1 ratio (BOX); 36 ppm lasalocid sodium (LAS; Taurotec^®^, Zoetis Inc., Campinas, São Paulo, Brazil); 30 ppm monensin sodium (MON; Bovensin^®^, Phibro Animal Health, Guarulhos, São Paulo, Brazil); 25 ppm virginiamycin (VIR; V-MAX^®^ 2, Phibro Animal Health, Guarulhos, São Paulo, Brazil); 30 ppm monensin sodium plus 25 ppm virginiamycin (VM); or 3.15% commercial mineral supplement with D-limonene and exogenous α-amylase (EOA; Fosbovi^®^ Confinamento CRINA^®^ RumiStar™, DSM S.A., Mairinque, São Paulo, Brazil).

The feed additives did not affect (*P* > 0.63) the rumen pH or temperature (Table 5). Also, no pH values below 5.2 were seen. The area under the curve and duration of pH above 5.8, and between 5.8 and 5.2 were not affected (*P* > 0.39) by the different feed additives.

**Table 5:** Effect of feed additive on pH and temperature of the rumen of feedlot Nellore bulls.

Item	BOX	LAS	MON	VIR	MV	EOA	SEM	*P*-Value
Mean pH^1^	6.27	5.96	6.05	6.03	6.01	5.95	0.000000266	0.634
Mean temperature, ˚C	39.7	39.6	39.8	39.7	39.8	39.6	0.11	0.676
Area pH, Δ pH × H								
pH > 5.6	15.0	7.00	11.2	10.9	8.71	9.82	2.582	0.392
5.6 < pH < 5.2	0.31	1.41	0.82	0.83	1.15	1.44	0.571	0.691
pH < 5.2	0.00	0.00	0.00	0.00	0.00	0.00	-	-
Duration pH, min/d								
pH > 5.6	1308	1089	1223	1161	1215	1129	139.4	0.887
5.6 < pH < 5.2	132	351	217	279	225	311	137.9	0.888
pH < 5.2	0.00	0.00	0.00	0.00	0.00	0.00	-	-

Diets were composed of 30% corn silage and 70% concentrate on a DM basis. The diets differed in the feed additive on a DM basis, as follows: 1.4% bicarbonate and magnesium oxide in 3:1 ratio (BOX); 36 ppm lasalocid sodium (LAS; Taurotec^®^, Zoetis Inc., Campinas, São Paulo, Brazil); 30 ppm monensin sodium (MON; Bovensin^®^, Phibro Animal Health, Guarulhos, São Paulo, Brazil); 25 ppm virginiamycin (VIR; V-MAX^®^ 2, Phibro Animal Health, Guarulhos, São Paulo, Brazil); 30 ppm monensin sodium plus 25 ppm virginiamycin (VM); or 3.15% commercial mineral supplement with D-limonene and exogenous α-amylase (EOA; Fosbovi^®^ Confinamento CRINA^®^ RumiStar™, DSM S.A., Mairinque, São Paulo, Brazil).

^1^The SEM of pH was expressed as H ion.

## Discussion

Supplementation with sodium bicarbonate may increase water intake, resulting in increased dilution of ruminal contents, rumen turnover, and consequently, greater intake [45–47]. In the current study, bulls fed BOX presented greater DM and OM intake compared to those fed the other diets. Likewise, other studies [47, 48] also reported that bulls fed BOX presented greater intake compared to those fed no additive or other feed additives, such as MON. However, responses to BOX on feed intake of beef cattle are variable [15]. For example, Zinn and Borques [49], reported a similar intake for animals fed diets with MON or BOX. The roughage source and greater forage inclusion in diets may increase chewing, rumination time, saliva production, and consequently, the endogenous buffering capacity in ruminants. Thus, such greater buffering capacity can reduce the effectiveness of dietary buffer supplementation, which may explain the variable response in feed intake when BOX is included in beef cattle diets.

Gouvêa et al. [13] and Meschiatti et al. [14] reported an increase in feed intake (7.4 and 8.6%, respectively) when MON was replaced by EOA. However, according to Gouvêa et al. [13], the roughage source may affect the DM intake when EOA is provided. These authors observed that when the roughage used was sugar-cane bagasse, bulls fed diets with EOA showed greater feed intake compared to those fed diets with MON. On the other hand, when the roughage was corn silage, the intake did not differ between EOA- and MON-based diets. These findings may justify our results, where a similar intake by bulls fed diets based on MON and EOA was observed.

According to Gorocica and Tedeschi [50], bulls fed MON and VIR diets presented similar feed intake, average daily gain, and feed conversion ratio. However, when adjusted for dose, bulls fed a diet with VIR presented greater average daily gain than those fed MON without affecting feed intake or feed conversion ratio. Other studies [19, 20] verified that feed intake and total tract digestibility were not affected by supplementation with MON, VIR or both. Moreover, according to Fonseca et al. [19] there were no differences in average daily gain or feed efficiency for bulls fed diets with MON, VIR, or both. Consequently, the inclusion of MV during the whole feedlot period may not justify the costs of its adoption. Other strategies, such as switching from MV to MON or VIR at some point during the feedlot period rather than feeding MV the whole time (e.g., adaptation period, finish phase, or daily) may be cost-effective [51–53].

Bulls fed diets based on MON or LAS presented similar intake and digestive parameters. Corroborating this, Berger et al. [54] suggest that animals fed diets with MON or LAS should present similar performance. However, different results are reported by NASEM [15], where the use of MON in feedlot diets can reduce feed intake by 3%, increase feed efficiency by 3.5%, and increase average daily gain by 0.52%, whereas LAS does not alter feed intake but increases feed efficiency by 3.5% and average daily gain by 3.63%.

In general, ruminal, and intestinal digestibility of most nutrients were similar among all feed additives, except for starch rumen digestibility. Bulls fed a diet based on EOA presented greater rumen digestibility of starch compared to the other diets. The potential for exogenous α-amylase to improve ruminal starch digestibility is reported in the literature [55, 56]. Therefore, exogenous enzymes have been used in ruminant nutrition to improve the efficiency of feed utilization, animal performance, and starch digestion [13, 14, 55]. Although greater starch rumen digestibility was presented by bulls fed EOA, total tract digestibility of starch did not differ among diets. Similar results were verified by Andreazzi et al. [55], where EOA improved ruminal starch digestibility but did not affect total tract digestibility.

Studies [47, 57] have reported an increase in the passage rate of rumen fluid and total rumen fluid for BOX-based diets compared to other feed additives. On the other hand, no results on total rumen fluid, dilution rate, or flow rate of rumen fluid for bulls fed diets with EOA are reported in the literature. Buffers and alkalizers are responsible for maintaining pH at a suitable level to promote the proper functioning of the rumen by neutralizing acidity through H^+^ sequestration and increasing the buffering capacity of ruminal fluid [58]. The increased rumen outflow may be beneficial for feedlot cattle consuming high-concentrate diets, since it can prevent severe oscillation in rumen pH.

Modern feedlot diets are characterized by high starch and minimum fiber concentrations to optimize animal performance. So, the feed additive needs to be effective in controlling the pH and avoiding metabolic disorders. In the current study, the mean rumen pH ranged between 6.0 and 6.3 for all diets. The lower area under the curve and lower duration of pH between 5.6 and 5.2, as well as the absence of rumen pH values below 5.2, suggest that the feed additives were efficient at controlling the ruminal pH and preventing acute and/or subacute acidosis [26, 59, 60]. Therefore, our findings suggest that all evaluated feed additives may be used in feedlot diets to minimize metabolic disorders, and consequently, decrease negative impacts on digestive parameters.

The rates of intake, digestion, and passage of DM, OM, apNDF, and starch were not affected by any of evaluated feed additives. Moreover, the results of this study suggest that no major differences in digestive parameters seem to exist for the evaluated feed additives. However, a performance study evaluating BOX LAS, MON, VIR, MV, and EOA supplementation should be conducted.

## Conclusion

The combination of sodium bicarbonate and magnesium oxide increased the nutrient intake compared to the other feed additives evaluated in the current study. The rumen pH, temperature, and kinetics did not differ between the evaluated feed additives. However, the addition of essential oils combined with exogenous α-amylase in the diet may increase ruminal starch digestibility and total tract DM and OM digestibility compared to the other feed additives.
